# Supplementary figures and images for: Higher bee abundance, but not pest abundance, in landscapes with more agriculture on a late-flowering legume crop in tropical smallholder farms
Source: PeerJ. 2021 Feb 19;9:e10732. doi: 10.7717/peerj.10732 (PMC7899018; doi:10.7717/peerj.10732)

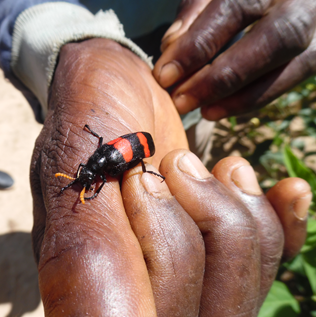

Supplement: Supplemental Information 6 — Photograph of the Hycleus spp. found feeding on pigeon pea flowers. Photo taken in May 2019 at one of the study sites. [file peerj-09-10732-s006.png]

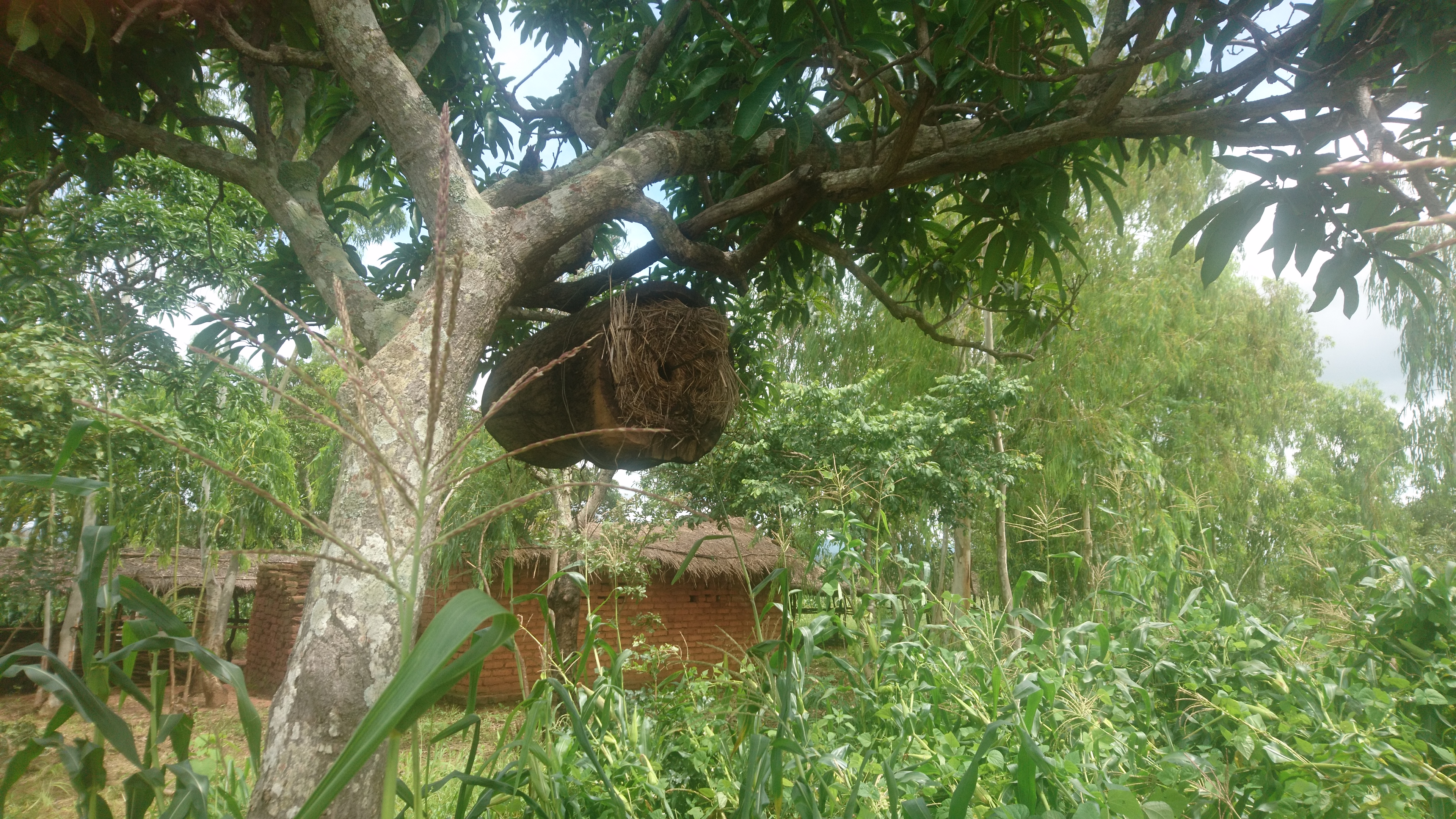

Supplement: Supplemental Information 7 — Photograph of a traditional honeybee hive as the farmers in our study area make them. They are hung in trees and not colonies are not actively placed in them –Farmers wait until they are colonized. The photo taken in February 2020 on a farm in Chiluzwazwa Ngwira, Mzimba district, Northern Malawi (not one of the study sites included in this study). [file peerj-09-10732-s007.jpg]

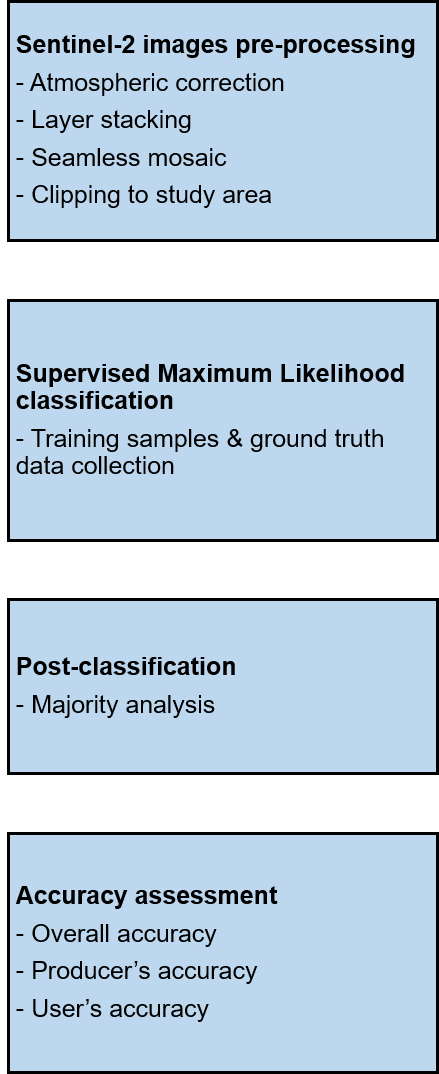

Supplement: Supplemental Information 8 [file peerj-09-10732-s008.png]

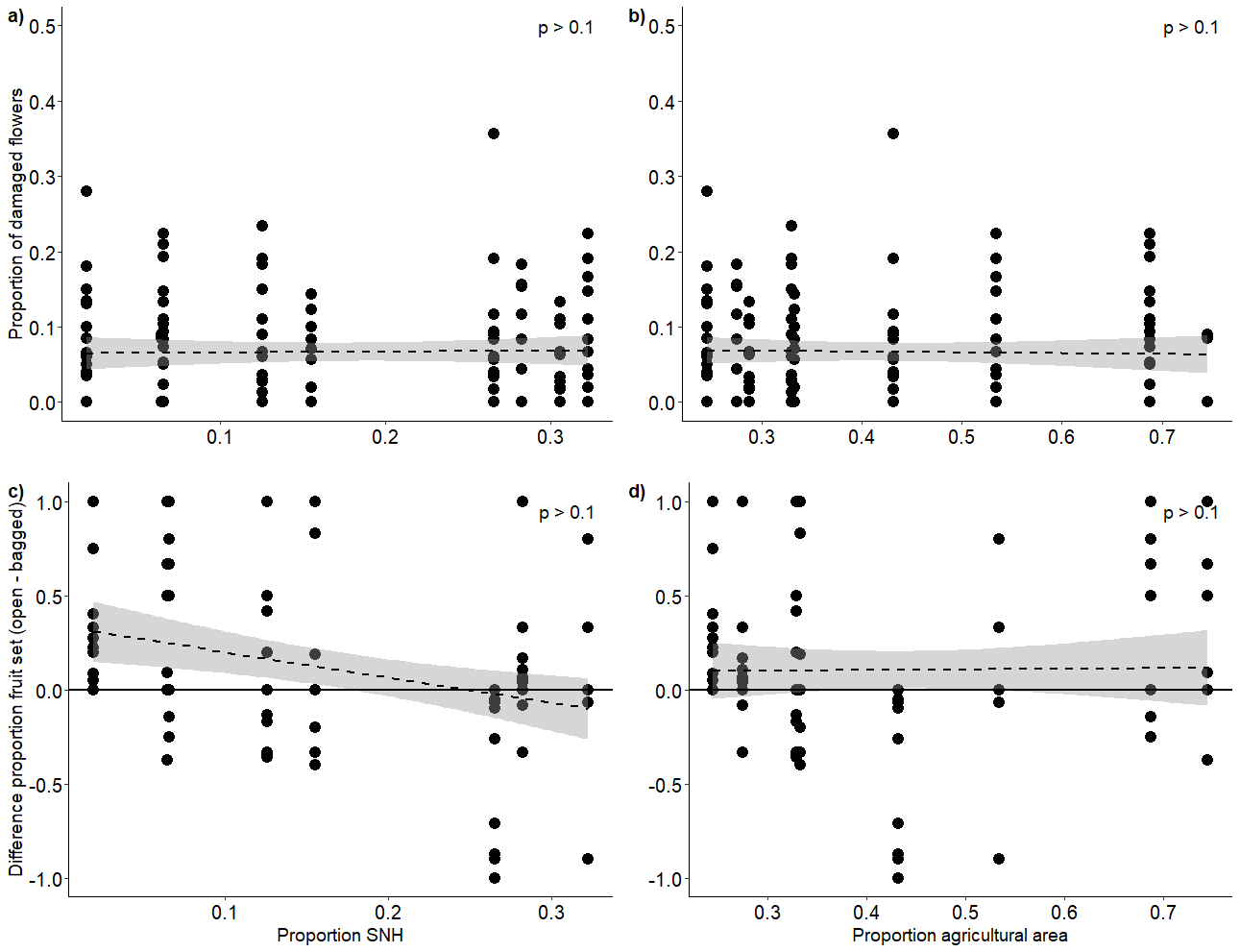

Supplement: Supplemental Information 12 — Relationship (±95% CI) between the proportion of semi-natural habitat on a) proportional flower damage and b) fruit set difference between clusters, as well as the proportion of agricultural area on c) proportional flower damage and d) fruit set difference between clusters. Points above the horizontal line indicate plants where the open clusters performed better than bagged clusters, points below the horizontal line indicate plants where bagged clusters performed better than open clusters. [file peerj-09-10732-s012.png]

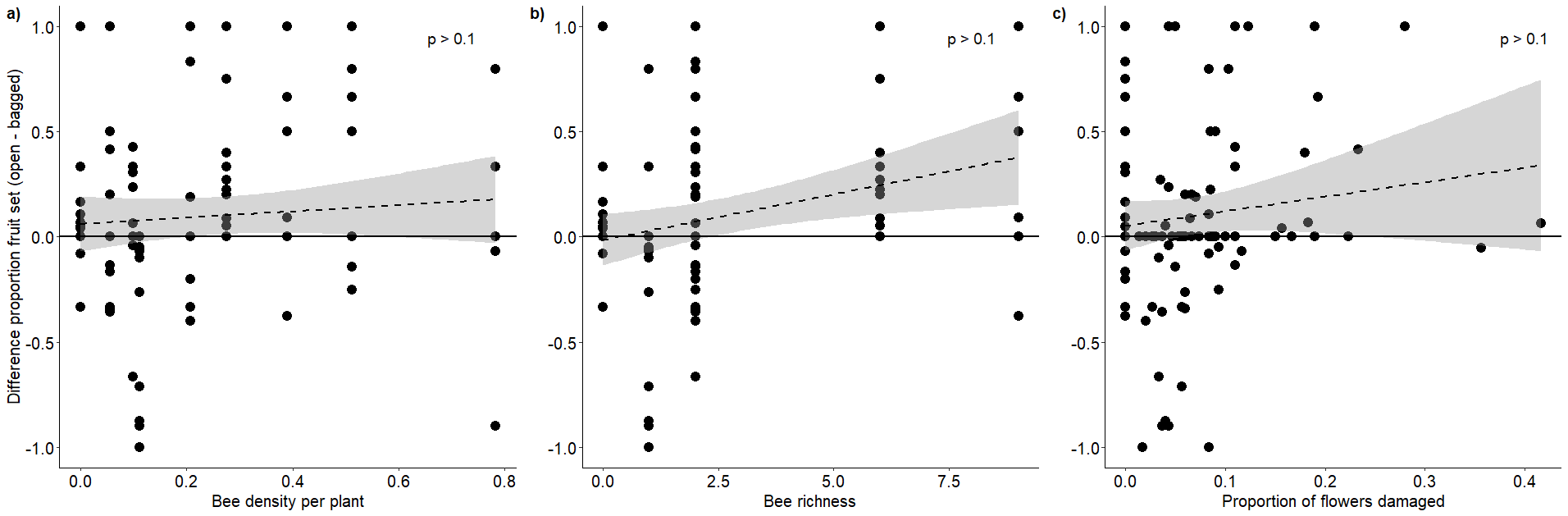

Supplement: Supplemental Information 13 — Relationship (+95% CI) between the difference in fruit set between open and bagged clusters and a) bee density, b) bee richness and c) proportional damage by blister beetles. Points above the horizontal line indicate plants where the open clusters performed better than bagged clusters, points below the horizontal line indicate plants where bagged clusters performed better than open clusters. [file peerj-09-10732-s013.png]

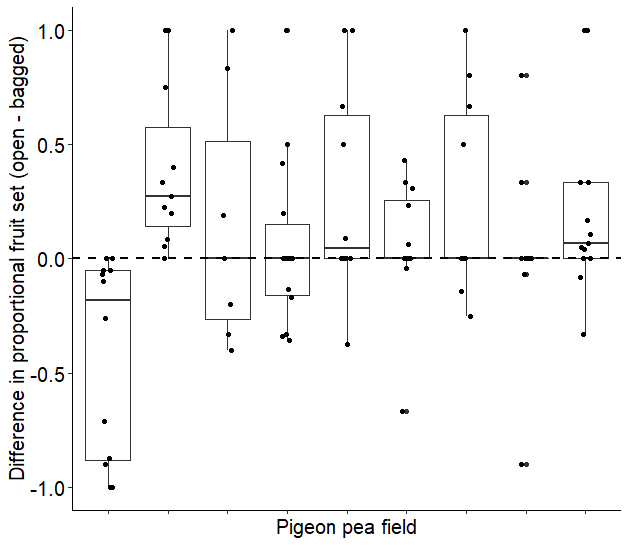

Supplement: Supplemental Information 14 — Fruit set difference between open and bagged clusters at the 9 sites where this was measured. Points above the horizontal line indicate plants where the open clusters performed better than bagged clusters, points below the horizontal line indicate plants where bagged clusters performed better than open clusters (n = 101). [file peerj-09-10732-s014.png]
