# Supplementary material for: Higher bee abundance, but not pest abundance, in landscapes with more agriculture on a late-flowering legume crop in tropical smallholder farms
Source: PeerJ. 2021 Feb 19;9:e10732. doi: 10.7717/peerj.10732 (PMC7899018; doi:10.7717/peerj.10732)
Supplement: Supplemental Information 9 [file peerj-09-10732-s009.docx]

| **Appendix 3:** Model summary of linear models assessing bee and blister beetle responses to planting density and field area (n=10) | | | | | | |
| --- | --- | --- | --- | --- | --- | --- |
| *Response* | *F-statistic (2,7)* | *Multiple R^2^* | *p-value* | *Predictor* | *t-value* | *p-value* |
| **Bee abundance** | 1.89 | 0.35 | 0.220 | **Shrubs per 75m** | -1.08 | 0.316 |
|  |  |  |  | **Field area** | 1.69 | 0.135 |
| **Bee richness** | 0.03 | 0.01 | 0.9681 | **Shrubs per 75m** | -0.12 | 0.912 |
|  |  |  |  | **Field area** | 0.24 | 0.82 |
| **Blister beetle abundance** | 1.81 | 0.34 | 0.233 | **Shrubs per 75m** | -1.388 | 0.208 |
|  |  |  |  | **Field area** | 1.39 | 0.206 |
