# Supplementary material for: Higher bee abundance, but not pest abundance, in landscapes with more agriculture on a late-flowering legume crop in tropical smallholder farms
Source: PeerJ. 2021 Feb 19;9:e10732. doi: 10.7717/peerj.10732 (PMC7899018; doi:10.7717/peerj.10732)
Supplement: Supplemental Information 10 [file peerj-09-10732-s010.docx]

| **Appendix 5:** Bee species collected across all sites and transects. Captured bees are stored in the Biocentre of the University of Würzburg. | | | |
| --- | --- | --- | --- |
| *Family* | *Genus (subgenus)* | *Species* | *Number of individuals* |
| Apidae | Apis | *A mellifera* (Linneus) | 52 |
|  | Xylocopa | *Sp1* | 3 |
|  |  | *Sp2* | 1 |
| Megachilidae | Megachile | *M. caricina* (Cockerel) | 1 |
|  |  | *Sp1* | 4 |
|  |  | *Sp2* | 2 |
|  |  | *Sp3* | 1 |
|  |  | *Sp4* | 6 |
|  |  | *Sp5* | 2 |
|  |  | *Sp6* | 5 |
|  |  | *Sp7* | 2 |
|  |  | *Sp8* | 1 |
| Halictidae | Nomia (Crocisaspedia) | *Sp1* | 4 |
| **Total** | | | **84** |
