# Supplementary material for: Higher bee abundance, but not pest abundance, in landscapes with more agriculture on a late-flowering legume crop in tropical smallholder farms
Source: PeerJ. 2021 Feb 19;9:e10732. doi: 10.7717/peerj.10732 (PMC7899018; doi:10.7717/peerj.10732)
Supplement: Supplemental Information 11 [file peerj-09-10732-s011.docx]

| **Appendix 6:** Model summary of linear models assessing the response of solitary and honeybee abundance responses to landscape composition (proportion semi-natural habitat and proportion agricultural area) (n=9). | | | | | | |
| --- | --- | --- | --- | --- | --- | --- |
| *Response* | *F-statistic (2,6)* | *Multiple R^2^* | *p-value* | *Predictor* | *t-value* | *p-value* |
| **Solitary bee abundance** | 1.45 | 0.33 | 0.506 | **SNH** | -1.39 | 0.240 |
|  |  |  |  | **Agricultural area** | 0.57 | 0.588 |
| **Honeybee abundance** | 1.91 | 0.39 | 0.228 | **SNH** | 0.70 | 0.510 |
|  |  |  |  | **Agricultural area** | 1.95 | 0.099 |
